# The Role of Tele-Exercise for People with Type 2 Diabetes: A Scoping Review

**DOI:** 10.3390/healthcare12090917

**Published:** 2024-04-29

**Authors:** Hani Fahad A. Albalawi

**Affiliations:** Department of Health Rehabilitation Sciences, Faculty of Applied Medical Sciences, University of Tabuk, Tabuk 71491, Saudi Arabia; hf_albalawi@ut.edu.sa

**Keywords:** diabetes, adult-onset diabetes, exercise, physical activity, digital health, telemedicine, tele-health, tele-rehabilitation, tele-physical therapy

## Abstract

Background: Supervised exercise interventions tend to be more effective than unsupervised exercises or physical activity advice alone. However, people with type 2 diabetes may find it difficult to attend supervised exercise interventions due to several obstacles. Tele-exercise, or utilizing technology to deliver home-based exercise, might be a solution. Objective: This scoping review aimed to explore clinical trials investigating the impact of tele-exercise interventions in individuals with type 2 diabetes Methods: Four electronic databases were searched for the period up to January 2024 for clinical trials investigating the impact of tele-exercise on health-related outcomes in adults with type 2 diabetes. Results: Seven trials involving 460 individuals with type 2 diabetes met the inclusion criteria. In these trials, combined aerobic and resistance exercise programs were the main types delivered remotely. To deliver such programs, both synchronous (n = 4) and asynchronous (n = 3) delivery modes were adopted. Regardless of the delivery mode, all tele-exercise interventions led to improvements in various factors related to type 2 diabetes and its complications, including glycemic control, blood lipids, body composition, functional capacity, muscle strength, and quality of life. The improvements were also found to be as effective as those of supervised exercise. Conclusions: Tele-exercise interventions seem to be feasible and as effective as supervised exercise interventions in terms of improving glycemic control, blood lipids, functional capacity, muscle strength, body composition, and quality of life for people with type 2 diabetes.

## 1. Introduction

Type 2 diabetes is a common chronic metabolic condition affecting people from all ethnic backgrounds [1]. According to the International Diabetes Federation, approximately 537 million people in the world have diabetes [2]. This number is also expected to increase to 783 million by 2045 [2]. Type 2 diabetes arises when the body’s insulin-sensitive tissues do not effectively respond to insulin, and there is insufficient production of insulin to compensate for this resistance [2]. Insulin is a hormone that functions to reduce blood sugar levels by promoting the absorption, utilization, and storage of glucose [3]. These issues in terms of insulin action and production result in elevated blood glucose levels, known as hyperglycemia [2].

Type 2 diabetes that is not properly managed can result in a range of complications, including cardiovascular disease, neuropathy, nephropathy, and retinopathy [1]. In addition, sarcobesity, i.e., the coexistence of decreased muscle mass and increased fat mass, is common among people with type 2 diabetes [4,5,6]. These complications increase the risk of disability and reduce the quality of life in people with this condition [7,8]. Previous studies revealed that, compared to people without the condition, people with type 2 diabetes tended to demonstrate poor performance on functional measures [6,9,10,11,12]. 

Exercise interventions are an essential component in the management of type 2 diabetes [13,14,15,16,17,18,19,20]. Both aerobic and resistance exercises induce cardiovascular and metabolic benefits in type 2 diabetes [13,14,15,16,17,21]. Aerobic exercises such as walking and cycling improve insulin sensitivity and glycemic control and reduce other cardiovascular risk factors, such as blood lipids, blood pressure, and body composition [22,23]. Resistance exercises, such as using free weights or elastic bands, provide significant advantages for glycemic control and offer further benefits in terms of increasing muscle strength and functional capacity [13,14,15,16,17,21]. However, studies comparing the impacts of different exercise interventions suggest that combined aerobic and resistance exercises are more beneficial than aerobic or resistance exercises alone [14,19,24,25]. 

The current type 2 diabetes guidelines recommend that individuals with type 2 diabetes engage in a minimum of 150 min of moderate aerobic exercise per week, along with two or three sessions of resistance training [18,26,27]. Balance exercises are also recommended, especially for those with physical limitations [18,26,27]. In addition, diabetes guidelines recommend the use of supervised exercise programs [18,27]. Studies examining the effects of supervised exercise found that such programs are more effective than either unsupervised exercise or physical activity advice alone [25,28,29]. Despite this, many people find it difficult to participate in supervised exercise due to various obstacles, such as pandemic restrictions, time constraints, and transportation issues. Therefore, effective strategies to facilitate the engagement of people with type 2 diabetes in supervised exercise programs are needed.

Recently, tele-exercise interventions, a subset of telerehabilitation, have emerged as a promising approach to overcoming barriers to exercise and improving health-related outcomes [30,31]. These interventions involve the delivery of exercise interventions remotely using the internet and telecommunication technology, enabling individuals to participate in supervised exercise from their own homes [30,31]. Tele-exercise interventions have demonstrated encouraging findings in terms of enhancing health-related outcomes in various chronic conditions [30,31,32,33,34,35]. For example, Tella et al. [35] found that tele-exercise interventions improved motor symptoms in people with multiple sclerosis [35]. In addition, a recent systematic review explored the effectiveness of tele-exercise interventions in various chronic conditions and reported that such interventions were feasible and effective in improving the physical capacity and quality of life [31]. However, of the 32 studies included in this review, only one study involved people with diabetes [31]. Therefore, the scoping review described in this paper seeks to investigate existing research exploring the impact of tele-exercise interventions in individuals with type 2 diabetes. It is anticipated that the results of this review will support healthcare providers, such as physical therapists, exercise physiologists, and primary care physicians, in adapting their clinical approaches to integrate tele-exercise for patients with type 2 diabetes.

## 2. Materials and Methods

A scoping review was conducted following the Preferred Reporting Items for Systematic Reviews and Meta-Analyses Extension for Scoping Review (PRISMA-ScR) guidelines [36]. This compromised the following five steps: (i) formulating the research question, (ii) identifying relevant studies, (iii) selecting appropriate studies, (iv) organizing the data, and (v) synthesizing, summarizing, and presenting the findings. 

### 2.1. Identifying Research Questions

Scoping reviews aim to synthesize evidence and evaluate the breadth of literature on a topic [37]. As such, three questions related to the aim of the review were established. These were as follows.

1. What are the types of exercise interventions delivered remotely through telecommunication technology for people with type 2 diabetes?
2. What are the telecommunication technologies used to deliver exercise programs for people with type 2 diabetes?
3. What are the effects of tele-exercise on health-related outcomes in individuals with type 2 diabetes?

### 2.2. Identifying Relevant Studies

To identify relevant studies, four electronic databases were searched from inception to January 2024 in consultation with a librarian. These databases were MEDLINE, PubMed, Web of Science, and the Physiotherapy Evidence Database (PEDro). The aim of the combined search of these subject-specific and multidisciplinary databases was to decrease the risk of missing eligible studies [38].

To guide the search strategy, the population, concept, and context (PCC) framework was used [39], where the target population was “ people with type 2 diabetes”, the concept was “exercise”, and the context was “tele-exercise”. Initially, an exploratory search was conducted in MEDLINE to explore the terminology used in the field. For this, the following keywords were used (type two diabetes AND exercise AND tele*exercise). Based on the results of this search, relevant search terms were extracted and categorized under the PCC categories. As such, keywords related to tele-exercise were expanded to include terms related to telehealth in general, such as tele-medicine and mobile health. Similarly, keywords related to exercise were expanded to include physical activity, as well as terms related to different types of exercise, such as walking, treadmill use, and free weights. Moreover, keywords related to diabetes were expanded to include terms such as diabetes and metabolic disease. 

The final keywords used in this review are presented in Table 1. Using these keywords, the final search was conducted across all included databases. Keywords for each category were combined together with the Boolean operator “OR”. The Boolean operator “AND” was then used to combine categories (Table 1). Manual citation index searches were conducted on related systematic reviews and included studies. 

### 2.3. Selecting Studies

All citations from the previous step were exported and imported into a web-based systematic review tool (www.covidence.org) to support the title and abstract screening process [40]. Then, the author independently proceeded with a two-step process. Firstly, the titles and abstracts of the identified articles were screened to evaluate their relevance to the research questions. Secondly, full-text articles underwent a review to ascertain whether they satisfied the inclusion criteria. 

To be included in this review, articles had to be randomized controlled trials (RCTs) or non-randomized controlled clinical trials (CCTs), focusing exclusively on adults with type 2 diabetes and written in English. The articles also had to evaluate an exercise program delivered remotely via technology and report at least one type 2 diabetes health-related outcome. For the purpose of this review, exercise was defined as a structured physical activity program, including activity types with specified intensities, frequency, and durations. Therefore, trials focusing on physical activity advice or self-management strategies were excluded. 

### 2.4. Charting Data

The data from the studies included were extracted independently by the author and organized using tables. The extracted data were the author(s), publication year, country, study design, duration of study, mode of delivery, type of technology used to deliver the intervention, features incorporated to facilitate delivery, exercise type, exercise intensity, exercise duration, intervention, diabetes-related outcomes, and findings. The data were then presented in two separate tables, provided in the Results section of this review.

### 2.5. Collating, Summarizing, and Reporting Results

The final step involved categorizing the relevant findings based on the research questions. Therefore, all findings were summarized and are presented in the Results section of this review.

## 3. Results

### 3.1. Characteristics of Selected Studies and Participants

**Figure 1:**
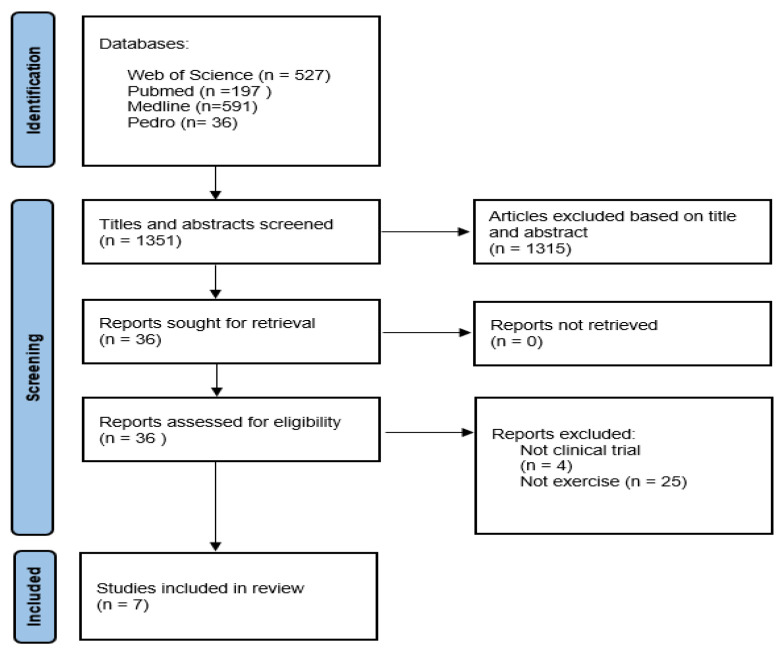
PRISMA diagram of the article screening process [36].

After removing duplications, the search strategies yielded a total of 1351 potential articles, of which 1315 were excluded based on the screening of the titles and abstracts (Figure 1). The remaining 36 articles were carefully read in full and assessed based on the inclusion and exclusion criteria. Finally, seven studies met the inclusion and exclusion criteria [41,42,43,44,45,46,47]. Of these, six studies were CRTs [42,43,44,45,46,47], and one was a CCT [41]. As shown in Table 2, the majority of the trials included were published recently: 2018 (n = 1) [42], 2019 (n = 1) [45], 2022 (n = 1) [47], and 2023 (n = 3) [43,44,46]. Only one study was published in 2008, in which electronic mail was used as a technology platform to deliver the exercise intervention [41]. 

The seven included trials involved a total of 460 adults with type 2 diabetes, with sample sizes ranging from 19 to 136 participants per study. These trials were conducted in various countries, including Saudi Arabia [43], the United States [41], Turkey [42,44,45,47], and Greece [46], with the majority having been conducted in Turkey [42,44,45,47]. Five trials recruited people with type 2 diabetes aged between 18 and 65. Two trials focused on older adults (i.e., >65 year) [41,44]. The duration since the diabetes diagnosis was reported in only three studies [42,44,45].

Only one study specifically recruited people with other comorbid conditions (e.g., mild dyspnea post-COVID-19 infection) [43]. The remaining six trials excluded people with other health conditions or comorbidities that could significantly impact their ability to participate in an exercise program, such as stroke, neuropathy, heart disease, and severe orthopedic conditions [41,42,44,45,46,47].

### 3.2. Characteristics of Tele-Exercise Programs 

Table 3 summarizes the characteristics of the exercise programs prescribed in the included trials. Overall, the exercise programs delivered remotely were short in length, ranging from 6 to 12 weeks, but generally adhered to the recommended amount of exercise for people with type 2 diabetes. In terms of the type of exercise, six studies prescribed a combination of aerobic and resistance exercises [41,42,43,44,46,47], while only one study prescribed resistance exercise alone in the form of callisthenic training [45]. 

Other types of exercise, such as balance and breathing exercises, were prescribed as an additional component in four studies [42,43,44,47]. Breathing exercises were prescribed in one study targeting people with type 2 diabetes and dyspnea post-COVID-19 infection [43]. Balance exercise was included as an additional component in three studies focusing on older adults with type 2 diabetes [41,44] or reporting physical capacity as an outcome [42,47].

In terms of intensity, all trials prescribed moderate-intensity exercises. In order to monitor the intensity, a perceived exertion scale and/or heart rate reserve using heart rate monitors was used in six trials [43,44,46,47]. One trial used both one repetition maximum for resistance training and a perceived exertion scale for aerobic exercise [41]. With regard to frequency, the exercise interventions were typically prescribed at a frequency of three times per week, with one study prescribing two sessions per week [41] and one study four times a week [43]. In terms of the duration of the exercise sessions, most studies prescribed sessions ranging from 30 to 60 min. 

### 3.3. Methods of Delivering Exercise Interventions

The exercise programs were delivered using both synchronous and asynchronous delivery modes. A synchronous mode, in which the exercise sessions were delivered instantaneously in real time, was utilized in four trials [43,44,45,46]. For this, the treating therapists met participants virtually using videoconferencing technology with audio and visual support (i.e., Zoom or Skype) [43,44,45,46]. This allowed for direct guidance and supervision from the treating therapist. 

On the other hand, three studies used an asynchronous mode in which pre-recorded exercise videos demonstrating the exercises and/or written materials describing the exercise were delivered to the participants [41,42,47]. The technology platforms used to deliver such interventions were electronic mail [41], websites [42], and mobile phone applications [47]. In this mode, the participants accessed the intervention at their convenience from a distance. To provide support and guidance to the participants, different features were adopted. These included text reminder messages, online communication with the treating physiotherapists if needed, self-tracking, heart rate monitoring used to adjust the intensity, verbal instructions, or written materials describing the exercises [41,42,47]. In addition, wearable devices to monitor the physical activity levels and provide feedback to the participants were utilized [47]. 

### 3.4. Impact of Tele-Exercise Interventions

The seven included studies compared the impact of tele-exercise interventions with usual care, active exercise interventions, or both and reported on a wide range of diabetes-related outcomes. Four RCTs with a two-arm design compared the effect of tele-exercise with that of either usual care [43,45,46] or an unsupervised exercise program [44]. Blioumpa et al. [46] examined the effect of a six-week tele-exercise intervention involving aerobic and resistance exercises on glycemic control, functional capacity, muscle strength, quality of life, and body composition in people with type 2 diabetes. The results showed that all outcomes improved significantly after the intervention as compared to the usual care group.

Duruturk and Özköslü [45] also explored the effect of a six-week tele-exercise intervention. In this study, the participants were randomly assigned to either usual care or a tele-exercise intervention involving resistance exercises in the form of callisthenic exercises. After six weeks, the results showed that, as compared to the baseline, only participants in the intervention group gained significant improvements in glycemic control, functional capacity, muscle strength, and psychological status. The improvements were also significant when compared to usual care, with the exception of glycemic control. Moreover, Nambi et al. [43] compared the impact of an eight-week tele-exercise program and usual care in people with type 2 diabetes following COVID-19 infection. Several outcomes were assessed before and after the interventions, including glycemic control, pulmonary function, functional capacity, and quality of life. At the end of the interventions, the results showed that tele-exercise was significantly more effective than usual care in improving all outcomes.

Furthermore, Tereks et al. [44] compared the effects of a six-week tele-exercise intervention and unsupervised exercise program. In this study, both groups were asked to perform a combined aerobic and resistance exercise program. The researchers then assessed the following outcomes: body composition, the ability to recover from stress using the Brief Resilience Scale, and quality of life. The results of this study demonstrated that all aforementioned outcomes improved significantly in the tele-exercise group and these improvements were statistically significant when compared to the unsupervised exercise group.

Two RCTs with three arms investigated the effect of tele-exercise as compared to both a control situation and another supervised active intervention [42,47]. Akinci et al. [42] compared the effect of an eight-week tele-exercise intervention to usual care and a supervised exercise intervention. The researchers found that, compared to usual care, both the tele-exercise and supervised exercise programs led to similar statistically significant improvements in glycemic control (HbA1c levels), functional capacity, blood lipid profiles, body composition, and quality of life. Timurtas et al. [47] explored the effects of two tele-exercise interventions (i.e., a mobile app group and mobile app plus wearable smartwatch group) as compared to a supervised exercise program. The results indicated that, as compared to the baseline, all interventions led to significant improvements in glycemic control, functional capacity, muscle strength, and pulmonary function. However, the difference between groups was not statistically significant in terms of the effects on these outcomes.

The remaining study was a CCT comparing the effect of a tele-exercise intervention delivered through electronic mail with that of an exercise program provided via printed materials [41]. In this study, both groups were provided with access to local fitness facilities to perform their prescribed exercises. After six weeks, the researchers noted that both interventions led to similar improvements in muscle strength and functional capacity in individuals with type 2 diabetes as compared to the baseline.

With regard to adherence and adverse effects, no study reported adherence rates or adverse effects specifically related to tele-exercise in individuals with type 2 diabetes. 

## 4. Discussion

This review aimed to explore existing tele-exercise interventions targeting people with type 2 diabetes. Overall, the evidence presented in this review was positive regarding the efficacy of tele-exercise in the management of people with type 2 diabetes. As compared to usual care, tele-exercise interventions were found to significantly improve a wide range of diabetes-related outcomes that can be classified into several domains, including glycemic control (i.e., HbA1c), blood lipid profiles, functional capacity, muscle strength, body composition, pulmonary function, and psychological status and quality of life. These outcomes are strongly associated with a number of complications associated with diabetes [48,49,50]. For example, people with poor glycemic control, as measured by HbA1c, were found to be at a greater risk of developing cardiovascular disease [49]. 

In addition, the findings suggest that tele-exercise interventions provide benefits comparable to those of traditional supervised exercise programs. In the current review, tele-exercise interventions were found to be as effective as traditional exercise interventions in improving health outcomes for individuals with type 2 diabetes [41,42,47]. This was consistent with previous studies exploring the efficacy of tele-exercise in people with other chronic conditions [51,52]. For example, a recent review evaluated the effectiveness of therapeutic exercise when it was tele-medically delivered and found that tele-exercise interventions were similar to face-to-face exercise interventions in terms of satisfaction, functional ability, and quality of life [52].

However, it should be noted that the components of exercise interventions play a critical part in achieving health benefits. In the current review, the majority of the studies incorporated moderate combined aerobic and resistance exercise programs at least three times a week, which adhered to the recommended level of physical activity for people with type 2 diabetes [18,27]. Only one study incorporated resistance exercises alone in the form of callisthenic exercises and did not show significant improvements in glycemic control [45]. Previous studies comparing the effects of different exercise programs found that combined exercise interventions were superior to either aerobic exercises alone or resistance exercises alone [24,53]. For example, Schwingshackl et al. reported that combined exercise training had a greater reduction in HbA1c levels compared to aerobic or resistance training alone in patients with type 2 diabetes [53]. In addition, previous studies indicated that the greater the amount of exercise, the more beneficial the effects of tele-exercise interventions.

The findings in the current review also support the use of both synchronous and synchronous modes of delivery. Studies utilizing either of these delivery modes reported positive results [41,42,43,44,45,46,47]. However, although both modes share the goal of providing exercise interventions remotely, they differ significantly in how they are delivered and implemented. Synchronous modes, being performed in real time, allow for immediate feedback and support, especially for those people requiring closer monitoring or motivation [54,55]. However, such a mode might be not useful for those individuals who have scheduling conflicts or are otherwise limited [54,55,56]. 

Asynchronous modes, on the other hand, provide flexibility in terms of when and where the exercise can be performed, allowing individuals to fit it into their own schedule [54,55,56]. However, there may be fewer opportunities for real-time motivation and guidance from the therapist [54,55,56]. To overcome such issues, the studies included in this review adopted several features, including text reminder messages, online communication with the treating physiotherapists if needed, self-tracking, heart rate monitoring used to adjust the intensity, verbal instructions, or written materials describing the exercises [42,45,47]. These features were recommended in previous research. For example, a recent review found that text message reminders can improve adherence to exercise programs, and online communication with healthcare providers can enhance support and guidance [57]. Furthermore, the use of self-tracking tools such as heart rate monitors can help individuals with type 2 diabetes to adjust the intensity of their exercises and ensure safety remotely [58]. 

Since both synchronous and asynchronous modes of delivery have their own advantages and disadvantages, the choice of the mode of delivery in tele-exercise interventions should take into consideration individual preferences and needs and the available resources [54,56,59]. According to the technology acceptance model, people come to accept and use a technology when they perceive that it is easy to use and useful [60]. Previous research has highlighted several factors that may influence the acceptability and successful implementation of tele-exercise, including technological proficiency, access to a reliable internet connection, and personal preferences [61,62,63]. Despite this, none of the studies included explained the degree to which the end-users were involved in designing the intervention, nor did they examine the acceptability of the intervention to the participants at the end of the intervention. Therefore, it is important for future studies to explore the end-users’ preferences and ensure that the chosen mode of delivery aligns with their needs and preferences.

### Clinical Implications, Limitations, and Future Research

To the author’s knowledge, this is the first scoping review focusing on tele-exercise for people with type 2 diabetes. The majority of the trials were published between 2018 and 2023, indicating a recent interest in this type of work. Therefore, the findings may help health professionals to make informed decisions about providing services to adults with type 2 diabetes. The available evidence shows that tele-exercise programs could be comparable to traditional supervised exercise programs in terms of improving glycemic control and other health outcomes, such as functional capacity and quality of life. However, it is important to acknowledge some limitations of this review. One is that there is limited literature exploring the use of tele-exercise for people with type 2 diabetes. This made it difficult to compare the effectiveness of different tele-exercise interventions. In addition, the majority of the studies reviewed were conducted in Turkey, making it challenging to draw definitive conclusions regarding the efficacy of tele-exercise in other countries. Furthermore, the small sample sizes in the studies could have affected the results of this review. Moreover, the acceptance of tele-exercise to the study participants was not investigated in the research reviewed. Therefore, it was impossible to draw a clear conclusion regarding the participants’ acceptance and satisfaction. Another limitation is that the search screening and data extraction were conducted by one researcher, and this may have led to bias. Future research in this area should focus on larger sample sizes, with participants from different ethnic backgrounds, to strengthen the evidence base for tele-exercise in type 2 diabetes management. Furthermore, additional research is needed to explore the acceptability of tele-exercise compared to traditional supervised interventions. 

## 5. Conclusions

Tele-exercise interventions seem to be feasible and as effective as supervised exercise interventions in terms of improving glycemic control, blood lipids, functional capacity, muscle strength, body composition, and quality of life for people with type 2 diabetes. However, there is a need for larger, more robust studies to confirm these findings, explore the preferences and needs of participants, and establish guidelines for the implementation of tele-exercise in type 2 diabetes management.

## Figures and Tables

**Table 1:** Search terms using the PCC framework.

Categories	Keywords with OR Boolean	Boolean to Combine Categories
Type 2 diabetes	type 2 diabetes OR type II diabetes OR diabetes OR diabetes mellitus OR metabolic disease OR none insulin dependent	AND
Exercise	exercise OR exercise therapy OR circuit-based exercise OR exercise movement techniques OR physical activity OR aerobic training OR aerobic exercise OR aerobic program OR circuit training OR circuit exercise OR swimming OR ergometer OR cycling OR bike OR running OR walking OR treadmill or resistance training OR resistance exercise OR strengthening training OR strengthening exercise OR thera*band OR balance training OR walking OR rehabilitation OR physiotherapy OR physical therapy	AND
Telecommunications	internet OR web OR web*based OR mobile application OR mobile health OR mobile medicine OR mHealth OR digital OR technology OR technology*based OR digital*health OR remote*medicine OR home*based OR zoom OR skype OR videoconference* OR tele*exercise OR tele*medicine OR Tele*health OR Tele*rehabilitation	AND

**Table 2:** Characteristics of the included studies.

Authors, Year of Publication	Study Design, Country	Participants	Intervention	Comparison/Control	Time Points (wk)	Outcome Measures	Changes in Outcomes
Duruturk and Özköslü [45] 2019	2-arm RCT Turkey	Total no: 50 (I: 25, C: 25) Age = 18–65 y T2D duration: >6 m Comorbidity: No Drop: 6 (I = 2, C = 4)	6 wk synchronous tele-exercise	Usual care	0–6	Glycemic control: HbA1c Functional capacity: 6MWT, sit-up, sit-and-reach test, back scratch, lateral flexion, and timed up and go Muscle strength: digital dynamometer, major upper/lower extremity muscles Psychosocial status and quality of life: Beck Depression Scale	HbA1c * All functional capacity outcomes ** Muscle strength ** Beck Depression Scale **
Akinci et al. [42] 2019	3-arm RCT Turkey	Total no: 65 (I = 21, C1 = 22, C2 = 22) Age: 40–65 y Duration of T2D: > 12 m Comorbidity: no Drop: 9 (I = 2), (C1 = 2), (C2 = 5)	6 wk synchronous tele-exercise	C1—usual care + brochure about lifestyle C2—supervised exercise	0–8	Glycemic control: HbA1c (%), FBG (mg/dL) Cholesterol: HDL, LDL, TG, TC (mg/dL) Body composition: BMI, WC (cm), HC (cm) Functional capacity: 6 MWT Psychosocial status and quality of life: EQ-5D	All outcomes improved ** compared to the control groups, not the supervised group Note: supervised and tele-exercise group had similar magnitude
Nambi et al. [43] 2023	2-arm RCT Saudi A rabia	Total no: 136 (C = 68, I = 68) Age: 18–60 y T2D duration: not reported Comorbidity: one month post-COVID-19 infection with mild dyspnea and T2DM Drop: 4 (C = 2, I = 2)	8 wk synchronous tele-exercise	Usual care	0–8–24–54	Glycemic control: HbA1c% Pulmonary function: FEV1, FVC, FEV1/FVC, MVV, PEF Functional capacity: six-minute walk test (6MWT) Psychosocial status and quality of life: (SF-12)	All outcomes ** at 8, 12, 24, 54 **
Taylor [41] 2008	2-arm CCT USA	Total no: 19 (C = 9, I = 10) Age: >65 T2D duration: not reported Comorbidity: no Drop: 2 (C = 1, I = 1)	6 wk synchronous tele-exercise	Printed materials, home exercise program	0–6	Muscular strength: upper and lower body parts (1-RM using leg and chest press) Functional capacity: O2 peak testing	All outcomes * No significant change between groups
Timurtas et al. [47] 2022	3-arm RCT Tukey	Total no: 90 C = 30, (I-A = 30, I-B = 30) Age: 30–65 T2D duration: not reported Comorbidity: no Drop: 6 (C = 3, I-A = 3, I-B = 4)	12 wk (2 asynchronous tele-exercise interventions)	Supervised exercise program	0–12	Glycemic control: HbA1c Functional capacity: 6MWT, sit and reach test, balance master system Muscle strength: isokinetic test LL, grip strength for UL using Jamar Pulmonary function: (FEV1), (FVC)	All outcomes improved in all groups * No significant change between groups
Terkes et al. [44] 2023	2-arm RCT Turkey	Total no: 70 (I = 35; C = 35) Age: >65 y T2D duration: 6 months Comorbidity: no Drop: 5 (I = 2, C = 3)	6 wk synchronous tele-exercise	Unsupervised exercise	0–6	Glycemic control: FBG Body composition: BMI Psychological status and quality of life: The Brief Resilience Scale, (CASP-19)	FBG * BMI ** The Brief Resilience Scale ** CASP-19 **
Blioumpa et al. [46] 2023	2-arms RCT Greece	Total no: 30 (I = 15; C = 15) Age: >40 y Diagnosis: T2D Duration: not reported Comorbidity: no Drop: 8 (I = 4, C = 4)	6-week synchronous tele-exercise	Usual care	0–6	Glycemic control: (HbA1c) Functional capacity: (6 MWT) Muscle strength: handgrip using dynamometer Body composition: BMI, HC, WC, waist/hip ratio Psychosocial status and quality of life: SF-36 Health Survey Questionnaire	All improved **

T2D = type 2 diabetes; I = intervention group; C = control group; M = male; F = female * = significant change pre–post; ** = significant change compared to control group; CCT: clinical non-randomized trial; RCT = randomized controlled trial; m = month; wk = week; FEV1 = forced expiratory volume in first second; FVC = forced vital capacity; MVV = maximum voluntary ventilation; PEF = peak exploratory flow; HbA1c (%) = glycated hemoglobin; FBG = fasting blood glucose level; BMI = body mass index; WC = waist circumference (cm); HC = hip circumference (cm) LDL = low-density lipoprotein; HDL = high-density lipoprotein; TC = total cholesterol; TG = triglyceride; 6MWT = six-minute walk test; EQ-5D = European Quality Of Life—5 Dimensions Questionnaire; SF-12 = Short-Form Health Survey-12; CASP-19 = Control, Autonomy, Self-Realization, and Pleasure.

**Table 3:** Characteristics of exercise programs.

	Delivery Mode	Technology	Type of Exercise	Intensity	Duration	Freq	Length	Features
Duruturk and Özköslü [45]	Synchronous	Videoconferencing system	Resistance + Breathing Exercises	Moderate (7 on Borg scale)	40 min	3/w	6 w	Online supervision
Akinci et al. [42]	Asynchronous	Website platform	Combined (Aerobic and Resistance) + Balance	Moderate, 7–11 on Borg scale	60–50 min	3/w	24 w	Phone messages Exercise videos with verbal instructions Online communications
Nambi et al. [43]	Synchronous	Videoconferencing system (Rehabapp)	Combined (Aerobic and Resistance) + Breathing	Moderate, 40–60% HR using app and the Borg scale	30 min aerobic 40 min resistance 10 breathing	4/w	8 w	Online supervision
Taylor [41]	Asynchronous	e-mail	Combined (Aerobic and Resistance)	Moderate using Borg scale and (1-RM)	20 min aerobic Resistance not reported	2/w	6 w	Printed instructions
Terkes et al. [44]	Synchronous	Videoconferencing system (Zoom)	Combined (Aerobic and Resistance) + Balance	Moderate, 60–65% of HR-Max.	50 min	3/w	6 w	Online direct supervision Heart rate monitor
Timurtas et al. [47]	Asynchronous	I-A (mobile app only) I-B (mobile app + wearable smartwatch)	Combined (Aerobic and Resistance) + Balance	Moderate, 40–60% HRR measured with an oximeter	30–60 min	w/3	12 w	Exercise reminders Online communication Wearable device to monitor heart rate and track activity
Blioumpa et al. [46]	Synchronous	Videoconferencing system (Skype)	Combined (Aerobic and Resistance)	Moderate (Borg scale)	60 min	w/3	6 w	Online direct supervision

w = week, Freq = frequency, HRR = heart rate reserve, I-A = first intervention A, I-B = second intervention.

## Data Availability

Not applicable.
